# Antibacterial activity of lactobacilli probiotics on clinical strains of *Helicobacter pylori*

**DOI:** 10.22038/ijbms.2019.33321.7953

**Published:** 2019-10

**Authors:** Parastoo Rezaee, Rouha Kasra Kermanshahi, Tahereh Falsafi

**Affiliations:** 1 Department of Microbiology, Faculty of Biological Sciences, Alzahra University, Tehran, Iran

**Keywords:** Antibacterial effect, CFS, Epithelial cell line, H. pylori, LAB, Urease activity

## Abstract

**Objective(s):**

Treatment of *Helicobacter pylori* infection by common drugs may be associated with several problems such as antimicrobial resistance to commonly used antibiotics and side effects of employed drugs. Therefore, exploration of non-chemical compounds which are safer than chemical ones is becoming important as an alternative therapy. The purpose of this study was to evaluate the effects of lactic acid bacteria (LAB) against clinical strains of *H. pylori*.

**Materials and Methods:**

Study of antibacterial effects of LAB against *H. pylori* strains included: evaluation of LAB effects as well as its cell-free supernatant (CFS) to reduce the number of *H. pylori*, and to examine the effects of CFS to inhibit the urease activity of *H. pylori*. The anti-adhesion effect of LAB on adherence of *H. pylori* to epithelial cell line was also evaluated.

**Results:**

Evaluation of the anti *H. pylori* effect of LAB depended on the strain of *H. pylori* and Lactobacillus. However, CFS of LAB reduced significantly the growth of all *H. pylori* strains. Also, urease activity of *H. pylori* strains was inhibited by CFS of LAB demonstrating that their organic acid may have a role in this inhibition. The significant anti-adhesion effect of LAB on adherence of *H. pylori* was also observed.

**Conclusion:**

Presence of LAB and/or their CFS can reduce the count of *H. pylori*, inhibit the urease activity of *H. pylori*, and reduce adhesion of *H. pylori* to epithelial cell line. This may be important for the impact of *H. pylori* colonization in the host stomach.

## Introduction

Bacterial interference between the natural flora and the pathogens was proposed in the 20^th^ century and following this finding, it was investigated for the control of infections. The investigators have demonstrated that the probiotics such as lactobacilli can prevent the growth of a wide range of human and animal microbial species which are their pathogens. Of them; the effects of Lactic Acid Bacteria (LAB) on *Helicobacter pylori *may be noted (1, 2). *H. pylori* has been known as a major cause of chronic gastritis, peptic ulcer disease and stomach cancer (3, 4). Bacterial virulence factors help *H. pylori* to invade the host stomach, cause disease and evade the host defenses. Expression of several virulence factors such as urease, adhesion factors, vacuolating cytotoxin A, and the cytotoxin-associated gene A, are associated with pathogenicity of* H. pylori* (5). The standard triple therapy regimen for treatment of *H. pylori* infection was widely used throughout the world (6). This treatment consisted of a proton pump inhibitor (PPI) plus two antibiotics (clarithromycin and amoxicillin) (7). The success of this treatment regimen has become progressively decreased in recent years. Non-compliance and the emergence of antibiotic-resistant strains of *H. pylori* are considered as the major factors contributing to treatment failure (8). Regarding the side effects of antibiotics in majority of the cases and emergence of resistant bacteria in the stomach of the infected patients, use of living microbial agents, such as LAB (as probiotics bacteria), may be a useful alternative in preventing the symptoms of *H. pylori* infection via inhibiting its growth by competing with this pathogens (9). Probiotics are definde as live microorganisms, which when administered in enough amount give a health benefit on the host. Probiotics have diverse mechanisms for inhibition of pathogenic bacteria, e.g. nutrient competition, production of inhibitory compound (bacteriocins, organic acids, biosurfactant,…) , immunostimulation and competition for binding sites (10).

The purpose of present study was to investigate the inhibitory effects of six species of LAB and their CFS against growth, urease (*in vitro*) and adhesion to epithelial cell line of eight clinical strains of *H. pylori *which were selected according to their diverse antibiotic resistance profiles.

## Materials and Methods

Bacterial strains and culture conditions: *H. pylori* strains used in this study were selected according to their susceptibility profile that were in *H. pylori* Lab at Alzahra University (Table 1). They were grown at 37 ^°^C for 48 hr under microaerobic conditions by Gas pack C (Merck) on Brucella Agar containing 5-10 % defibrinated sheep blood supplemented with vancomycin (10 μg/ml), polymyxin (2.5 IU/ml) and amphotericin B (2 μg/ml). Antibiotic susceptibility testing of *H. pylori* strains was performed according to agar disk-diffusion procedure (11). For this purpose, bacterial suspension (McFarland tube No 4) corresponding to approximately 9×10^8 ^CFU/ml were plated on Muller Hinton Agar (MHA) (Merck) containing 5% defibrinated sheep blood without antibiotic. Antibiotics disks corresponded to amoxicillin [25 µg/disk], tetracycline [30 µg/disk], ampicillin [10 µg/disk], ciprofloxacin [5 µg/disk], azithromycin [15 µg/disk], erythromycin [15 µg/disk], ceftriaxone [30 µg/disk], cefixime [5 µg/disk], furazolidone [100 µg/disk], Metronidazole (5 µg/disk) and Clarithromycin (15 µg/disk) purchased from Padtan Tab were placed on the plates and incubated at 37 ^°^C in a microaerobic condition for 72 hr, then their inhibition zone diameters were examined. The zones of inhibitions were interpreted according to those of the previously described protocol (12-15).

LAB corresponded to *Lactobacillus acidophilus* ATCC 4356*, Lactobacillus rhamnosus* ATCC 7469, *Lactobacillus reuteri* ATCC 23272, *Lactobacillus fermentum* ATCC 9338, *Lactobacillus plantarum* ATCC 8014 and *Lactobacillus casei* ATCC 39392 that were provided from Iranian Research Organization for Science and Technology (IROST). These LAB were selected since their antibacterial effects on the most photogenic bacteria has been confirmed in our previous researches (10). LAB were cultured in DeMan-Rogosa-Sharpe (MRS) broth for 48 hr at 37 ^°^C. CFS of LAB were prepared from their 48 hr culture by centrifugation at 10000×g for 10 min at 4 ^°^C, and by filtration through the 0.22-μm-pore-size filter (Millipore).

**Figure 1:**
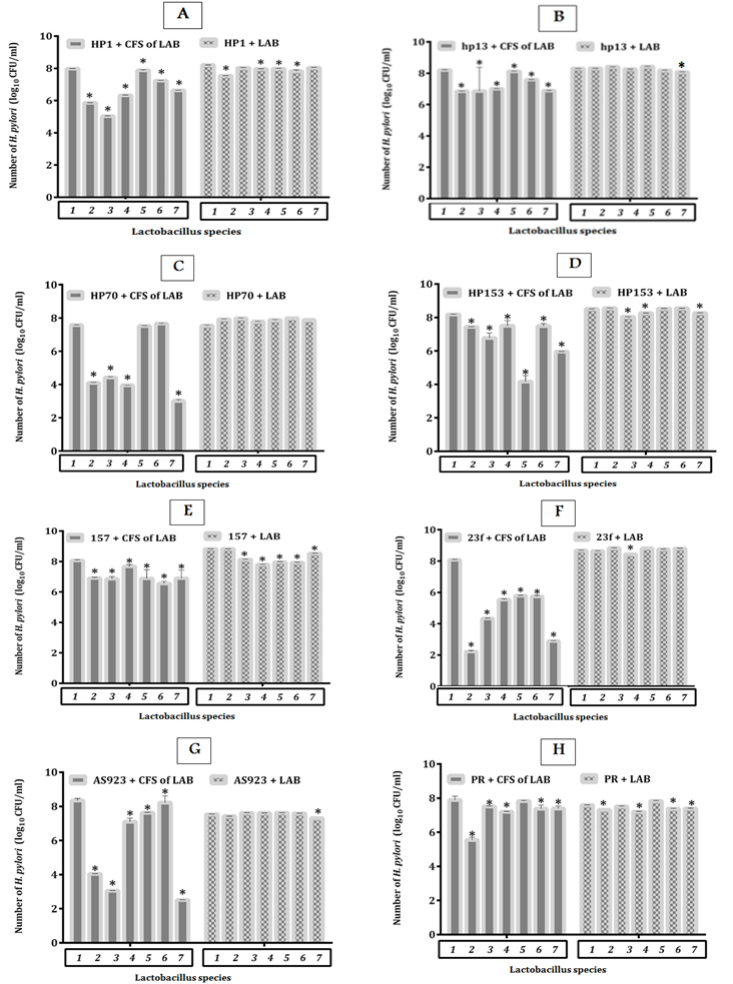
(A-H) Count of *Helicobacter pylori* strains (log_10_ CFU/ml) in presence of cell free supernatant (CFS) of lactobacillus bacteria and lactobacillus bacteria alone after 48 hr incubation at 37 ^°^C (equal volume, initial concentration of bacteria were 10^8^ CFU/ml). (1: MRS Broth Medium; 2: *Lactobacillus acidophilus* ATCC 4356; 3: *Lactobacillus rhamnosus* ATCC 7469; 4: *Lactobacillus reuteri* ATCC 23272; 5: *Lactobacillus fermentum* ATCC 9338; 6: *Lactobacillus plantarum* ATCC 8014; 7: *Lactobacillus casei* ATCC 39392)

* indicate significant differences from the control (MRS Broth Medium) by T-Test (*P<*0.05)

**Figure 2:**
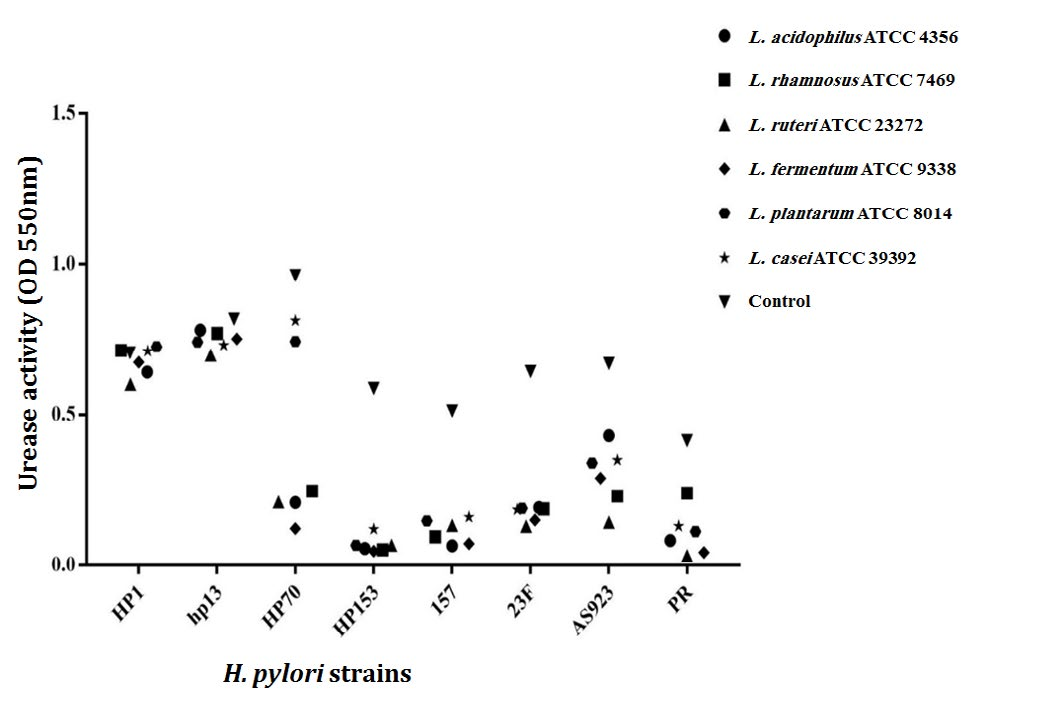
Effect of lactobacilli cell free supernatant (CFS) (30% concentration) on urease activity of *Helicobacter pylori* strains by measuring the absorbance of ammonium at 550 nm after 120 min

Differences with a *P- value* below 0.05 were considered statistically significant by paired T-Test

**Figure 3:**
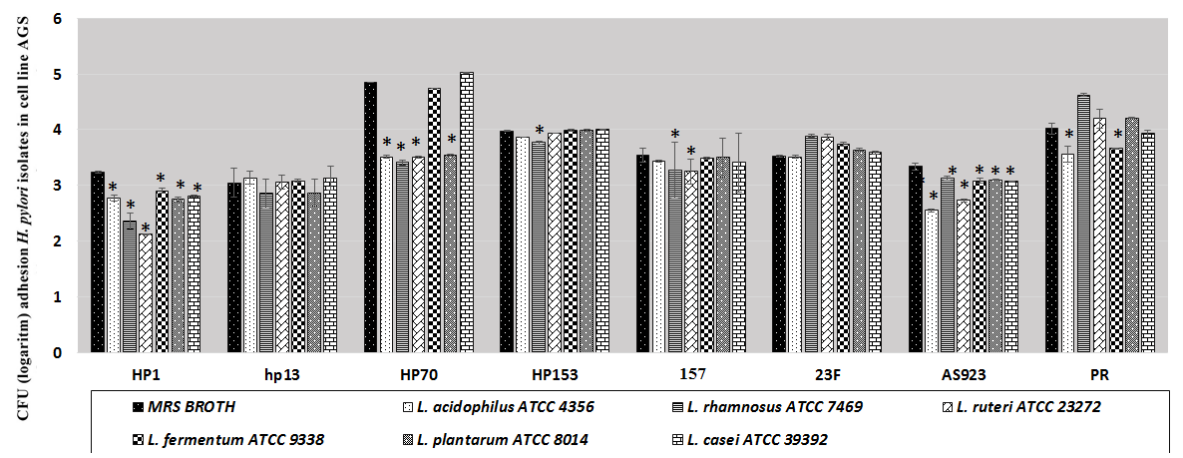
Number (log_10_ CFU/ml) of adherent *Helicobacter pylori *on AGS cell line in the presence and in absence of lactobacillus bacteria after 3 hr incubation at 37 ^°^C. Data represent mean±standard deviation (SD) from 3 independent experiments (error bar)

*indicate significant differences from the control (MRS Broth Medium) T-Test (*P<*0.05)

**Table 1:** *Helicobacter pylori* strains used in this study

	*H. pylori *strains	Antibiotic resistance profile		Sex-age- pathalogy
		Resistance	Rensitive	Male-14-CG
1	HP1	Met, Cly, Amox, Amp, FR, E, ATH	TE, CIP, CRO, CFM	Female-13-CG
2	hp13	Met, Cly, Amox, Amp, TE, E, ATH	CIP, FR, CRO, CFM	Female-62-CG
3	HP70	Met, Cly, Amox, Amp, TE, E, ATH	FR, CRO, CFM	Female-24-CG
4	HP153	Met, Cly, Amox, Amp, TE, FR, E, ATH, CFM	CIP,CRO	Female-4-CG
5	157	Met, Cly, Amox, Amp, FR, E, ATH	TE, CIP, CRO, CFM	Male-9-CG
6	23F	Met, Cly, Amox, Amp, TE, CIP, E, CFM, CRO, FR, ATH	-	CG
7	AS923	Met, Cly, Amox, Amp, CIP, E, FR, E, ATH	TE, CRO, CFM	Male-4-CG
8	PR	Met, Cly, Amox, Amp, TE, CIP, CFM, CRO, FR, E	ATH	Male-14-CG

CG: Chronic Gastritis

Amo: Amoxicillin (25 µg/disk), TE: Tetracycline (30 µg/disk), Amp: Ampicillin (10 µg/disk), CIP: Ciprofloxacin (5 µg/disk), ATH: Azithromycin (15 µg/disk), E: Erythromycin (15 µg/disk), CRO: Ceftriaxone (30 µg/disk), CFM: Cefixime (5 µg/disk), FR: Furazolidone (100 µg/disk), Met: Metronidazole (5 µg/disk), Cly: Clarithromycin (15 µg/disk)

**Table 2:** Reduction (%) of *Helicobacter pylori* in presence of Cell Free Supernatant (CFS) & lactobacillus bacteria

LAB	Strains of *H. pylori *% (n=8)	
	CFS	LAB
*L. acidophilus *ATCC 4356	100% (8)	25% (2)
*L. rhamnosus *ATCC 7469	100 %(8)	25% (2)
*L. ruteri *ATCC 23272	100 %(8)	62.5% (5)
*L. fermentum *ATCC 9338	50% (4)	25% (2)
*L. plantarum *ATCC 8014	75 %(6)	37.5% (3)
*L. casei *ATCC 39392	100 %(8)	62.5% (5)

***Cell line***

The gastric epithelial cell line AGS (ATCC CRL-1739) was cultured in RPMI 1640 (Dacell) supplemented with 10% heat-inactivated fetal bovine serum (Gibco) and 1% PenStrep (100 U/ml penicillin and 100 µg/ml streptomycin, bioidea). The cells were maintained at 37 ^°^C and 5% CO_2_ in a humidified environment. The day before of experiment, the cells were seeded into tissue culture plates to form a monolayer overnight. At the start of each experiment, the cell culture medium was replaced with RPMI 1640 with 3% serum and without antibiotics.

***Effect of lactobacilli CFS on H. pylori***

*Agar diffusion assay*

*H. pylori* suspensions equivalent to 2 McFarland, were plated on Mueller-Hinton agar plates containing 5 % defibrinated sheep blood without antibiotics. Wells (diameters, 6 mm) were drilled into the agar using the sterile Pasteur pipettes. They were filled with 100 μl CFS of LAB. Plates were incubated for 72 hr under microaerobic conditions at 37 ^°^C, and the diameters of inhibition zones around the wells were measured according to the previously described protocol (16). 

*Liquid culture assay*

*H. pylori* suspension (10^8^ CFU/ml) were prepared in brain heart broth without antibiotics and were incubated under microaerobic conditions at 37 ^°^C with CFS (equal volume); MRS broth medium was used as the control. The viability of *H. pylori* after 48 hr was evaluated by determination of the viable count (CFU) on MHA agar containing 5% defibrinated sheep blood plates following incubation at 37 ^°^C under microaerobic conditions (16). 

***Effect of lactobacilli CFS on urease activity of H. pylori strains***

*The urease activity of H. pylori strains*

Urease activity was determined in medium containing, phosphate buffer, urea and 0.012% phenol red (as indicator of pH). The principle of this analysis was the production of ammonia due to hydrolysis of urea, which was measured by absorbance of produced color at 550 nm by the spectrophotometer. Optimization of the reaction conditions concerning urea concentration, pH of reaction buffer and temperature for urease activity of *H. pylori* was performed before analysis of urease activity. For this purpose, the optimum effect of urea concentration (15 to 25 mg/ml), pH (5.8 to 7.8 pH), and temperature (37 to 55 ^°^C) was examined by response surface methodology (RSM) (17). In all cases, 50 ul of *H. pylori* cells, grown in the Brucella broth for 48 hr at 37 ^°^C (initial number of bacteria: 10^8 ^CFU/ml) was added to 1 ml of urease reaction buffer and the color absorbance was measured at 550 nm after incubation for 1 hr (18-20).

*The inhibitory effect of lactobacilli CFS on urease activity of H. pylori*

For analysis of urease inhibitory effects by LAB, *H. pylori *cells incubated in Brucella broth at 37 ^°^C (initial number: 10^8^ CFU/ml) for 48 hr, were mixed with various concentrations of CFS (10 to 40%) and incubated at 37 ^°^C for 30, 60, and 120 min. The resulted color was read at 550 nm by spectrophotometer according to the previously described protocol (16). MRS broth was used as control of reaction. Also, the effect of CFS that could be neutralized by NaOH (1 mol/lit) to 7.0±0.01 and acids such as acetic acid (Merck) and lactic acid (Merck) on urease activity were evaluated (21). 

***Effect of LAB on the count of H. pylori strains by co-culture***

*H. pylori* strains also LAB were recovered from plates and were suspended in BHB with 10 % (v/v) horse serum and MRS broth, respectively (McFarland No. 1 and 0.5 respectively). Equal volumes of each suspension was combined and incubated at 37 ^°^C at atmosphere containing 5% CO_2_. After 48 hr incubation at 37 ^°^C under microaerobic conditions, the number of CFU was evaluated on MHA agar containing 5% defibrinated sheep blood which was selective for the growth of *H. pylori* containing 5 µg/ml trimethoprim, 2.5 U/ml polymyxin B, 10 µg/ml vancomycin and 5 µg/ml penicillin (Sigma-Aldrich) (the LAB were sensitive to penicillin but *H. pylori* was resistance)(22). 

***Effect of LAB on adhesion of H. pylori to AGS cell line***

Bacterial suspensions were prepared from the culture plates of* H. pylori* strains and adjusted to McFarland tube No 3. After centrifugation, bacterial cells were suspended in RPMI 1640 with 3% serum. As well, bacterial suspensions were prepared from the overnight culture plates of LAB and adjusted to McFarland tube no 0.5; after centrifugation, bacterial cells were suspended in RPMI 1640 with 3% serum. Epithelial cells grown in 48-well plates were infected with both *H. pylori* strains and LAB at an multiplicity (MOI) of 100 for each species. After 3 hr of incubation, the cells were washed three times with phosphate-buffered saline (PBS) to remove any unbound bacteria. The host cells were lysed by treatment with Brucella broth containing 10% fetal bovine serum (FBS) for 30 min, and then all cells from the wells were scrubbed. The number of CFU for *H. pylori* was determined by serial dilution and spreading on appropriate selective medium as previously described after incubation for 4 to 7 days (23).

***Statistical analysis***

All of the experiments were performed in triplicate samples and error bars represent standard deviations. Differences with a *P-*value below 0.05 were considered statistically significant by paired T-Test. Normality of the data and the statistical analysis was performed using Minitab 17.

## Results

***Effect of LAB and their CFS on the count of H. pylori***

The count of *H. pylori* strains (Log_10 _CFU/ml) in the presence of CFS of LAB and LAB are shown in Figure 1 (A-H). Results demonstrated that the reduction effect of CFS was higher than LAB. In the case of CFS was between 1-6 log but in the case of LAB was not more than 1 log. In the case of CFS of *L. acidophilus* ATCC 4356 and *L. casei* ATCC 39392 a reduction of 6-log was observed in *H. pylori* 23F but only a reduction of 1 log was observed in case *H. pylori* 23F in presence of *L. ruteri* ATCC 23272. 

Based on Figure 1 (A-H), CFS of *L. acidophilus, L. rhamnosus, L. reuteri* and *L. casei *had a decreasing effect on the growth of every 8 strains of *H. pylori* but *L. fermentum* and *L. plantarum* had this effect on the growth of 4 and 6 strains of *H. pylori* respectively. Also this effect on LAB included 5 strains of *H. pylori *in presence of* L. ruteri *and* L. casei, *3 strains in presence of *L. plantarum* and 2 strains in presence of other LAB (Table 2). 

***Effect of lactobacilli CFS on the urease activity of H. pylori***

To perform this test, the optimized conditions for urea concentration, pH of reaction buffer and temperature were obtained using RSM method. They were 15 %, 5.8 and 46-55 ^°^C for urea concentration, pH, and temperature, respectively. The anti-urease activity effect of CFS was higher at concentrations of 30-40% than other concentrations, but the same effect was observed in these two concentrations. Figure 2 shows the results of the anti-urease activity effect of CFS in the concentration of 30% after 120 min. The result showed that CFS of *L. acidophilus* ATCC 4356, *L. reuteri *ATCC 23272 and *L. fermentum* ATCC 9338 reduced urease activity of all eight *H. pylori* strains. However, *L. rhamnosus* ATCC 7469, *L. plantarum* ATCC 8014 and *L. casei* ATCC 39392 were effective only on urease activity of six out of eight *H. pylori* strains. This inhibition effect was lost in pH of 7.0, indicating that this effect may be mediated by organic acid present in CFS. As the control, we tested the effect of various concentrations of acetic acid and lactic acid (10, 20 and 30%) on urease activity of *H. pylori *and we obtained similar results as above. The most effective inhibitory effect was observed for *H. pylori* HP70 such that its initial absorbance of 0.963 at 550 nm was changed to 0.122, 0.2 and 0.208 after 120 min in presence of CFS obtained from *L. fermentum* ATCC 9338, *L. reuteri* ATCC 23272 and *L. acidophilus* ATCC 4356 respectively (Figure 2).

***Effect of LAB on the adhesion of H. pylori to AGS cell line***

By counting the number of adherent *H. pylori* to host cell AGS, the percentage of adherence was analyzed. We observed that adherence in the *H. pylori* HP1 and AS923 (20%) were inhibited by all of the LAB. However, *L. rhamnosus* ATCC 7469 had anti-adherent effect against five *H. pylori* strains, and *L. acidophilus* ATCC 4356 and *L. reuteri* ATCC 23272 had anti-adherent effect against four *H. pylori* strains. Adherence of *H. pylori* HP1 and AS923 was reduced in presence of all LAB but in the cases of *H. pylori* hp13 and 23F no decrease was found. Figure 3 showed CFU (log_10_/ml) of adherent bacteria to AGS in the presence and in absence of LAB.

## Discussion

Treatment of *H. pylori* infection is often effectuated by routine triple therapy regimen and if the infecting strain is resistant, its successful eradication would be compromised (24). Nowadays, the rate of treatment failures is rising and the major cause for this would be bacterial resistance to frequently prescribed antibiotics, so, it is important to know the pattern of this resistance. Furthermore, the antibiotic resistance patterns vary between countries and within different regions of the same country (25, 26). The major pattern of antibiotic-resistance in *H. pylori* strains used in the present study was resistance to metronidazole, clarithromycin, amoxicillin, ampicillin, erythromycin, furazolidone, and azithromycin (Table 1). Studies of researchers in Iran recommended fluoroquinolones for the treatment of *H. pylori* infection because of the inadequacy of the common antibiotics (27, 28). This resistance pattern reﬂects the importance of antibiotic use in our country, especially in children (11). The results of a study on 218 isolates of *H. pylori* gathered from 985 dyspeptic patients during 2010-2017 in Iran, showed a considerable increase in resistance to presently used antibiotics such as metronidazole, ofloxacin, tetracycline, and clarithromycin (29). This high resistance to antibiotics, requires alternative ways to reduce the rates of *H. pylori *infection 

Probiotics bacteria are prescribed for the treatment of many gastrointestinal disorders, ranging from diarrhea to *H. pylori* infection (15). Researchers demonstrated an increase in the rate of *H. pylori* eradication due to the use of probiotics along with triple therapy (30). The *in-vivo* studies have shown that eradication rate of *H. pylori* infection by drugs can be improved by administration of lactobacilli and also due to reducing gastric mucosal inflammation (31-34). The decrease of 6-log of *H. pylori* was reported in presence CFS of *L. fermentum* after 24 hr (35). Complete inhibition of *H. pylori* *in vitro* was observed in the case of co-incubation with probiotics (*L. rhamnosus* and *L. acidophilus*) at ratios of 1:10 and 1.100 (36). Takeda *et al.* reported that inhibitory activity of probiotic bacteria against *H. pylori* was strain-dependent so that *L. paracasei* strain 06TCa19 and *L. plantarum* strain 07MR044 exhibited potent abilities to inhibit the growth of *H. pylori* in co-culture method (22), that detected in the present study. Antagonistic effect of LAB related to resource competition, production different low molecular weight substances (e.g. diactyl, acetaldehyde, hydrogen peroxide, ect.); production of different organic acid (e.g. lactic, propionic, succinic and acetic); pH lowering effect and production of bacteeriocin and bacteriocin-like substances that are produced differently by LAB so the inhibitory effects of LAB on *H. pylori* differ from strain to strain (37, 38).

In the present study, we used the LAB of human origin that has different metabolism pathway (fermentation pathways). Our results demonstrated that both CFS of LAB and LAB displayed anti-*H. pylori* effect but CFS had more effect than LAB. Both CFS and LAB caused a significant reduction in the growth of two *H. pylori* strains (23F and PR) which were highly resistant to antibiotics. Reducing the number of colonized bacteria may also be important in Quorum sensing (QS) process which is a regulatory mechanism used by bacteria to receive and respond to variations in cell-population density. This is effectuated through the expression of specific genes (39). In the *H*. *pylori* genome, the only known QS gene is the *luxS* gene. *LuxS* has an alternative role in the regulation of motility (by modulating flagellar transcription and flagellar biosynthesis) and biofilm formation (40). The expression of this gene may be altered by decreasing the number of *H. pylori* which then can alter the regulation of motility and biofilm formation.

Higher anti-*H. pylori *effect of CFS observed may be due to the fact that CFS contains antimicrobial agents that causing death of *H. pylori* during the early growth time. The anti- *H. pylori* effect of CFS was lost or decreased when the pH of CFS adjusted to 7 (the data not showed). In a genotobiotic murine model, *L. salivarius* produces high levels of lactic acid and thereby inhibits growth of *H. pylori*, it has been suggested that the concentration of organic acids was related to anti- *H. pylori* activity of CFS of LAB (41,42). Inhibition of *H. pylori* by the production of lactic acid in *L. salivarius*, *L. acidophilus, L. rhamnosus *and* L. casei* strain Shirota has been reported (43), although was demonstrated that anti-inhibition effects of Lactobacillus strains were only partly explained by organic acid production (44). Coconier *et al.* showed that supernatant of *L. acidophilus* decreases the survival ability of *H. pylori* due to the presence of anti-Helicobacter substances produced by *L. acidophilus* that may be different from lactic acid (45). Different studies have used agar well diffusion assay to determine CFS susceptibility of *H. pylori* (16, 37) but the results of our experiment, were not measurable due to the small diameters of inhibition zones. 

Our results concerning CFS effect against urease activity of *H. pylori* strains showed that CFS of all LAB could reduce urease activity of *H. pylori* HP70, HP153, 157, 23F, AS923 and PR during 120 min. Urease activity is essential for initiating the stomach colonization of stomach by *H. pylori* which posses both cytoplasmic and surface-associated or extracellular urease and activity of surface-localized urease is essential for resistance of *H. pylori* to acid (46). In our study, CFS of probiotics bacteria could decrease urease activity but no inhibitory effect was found on urease activity when pH of CFS was neutralized with NaOH. Therefore, organic acids in CFS of LAB play an important role in the inhibition of urease activity of *H. pylori* strains. Also, inhibition effect of urease by acetic acid and lactic acid was observed in our work. The urease activity of *H. pylori* co-cultured with lactobacillus supernatants decreased (20). Lactic acid of *L. femntum* UCO-979C obtained from human gut could inhibit the urease enzyme of *H. pylori* strains (35). However, the bacteriocin of *L. plantarum* and *L. acidophilus* could inhibit or reduce the urease activity in Proteus spp (47).

 Inhibitory effects of LAB on adherence of *H. pylori* strains to AGS cell line may reflect their *in vivo* effect that can help to prevent infection in an early stage of *H.* *pylori *colonization. The anti-adherence effects of probiotics can be produced by bacterial competition for binding sites on epithelial cells where at the same time the antimicrobial substances are also secreted by probiotic bacteria. *L. reuteri* possesses the cell surface proteins that inhibit *H. pylori* to bind to glycolipids receptor *in vitro* (48), also lactobacilli act directly on *H. pylori* by an effectors molecule that is released into the medium. This effectors molecule acts on *H. pylori* by inhibiting expression of the adhesion-encoding gene* sabA* (23). Animal studies demonstrated that prior colonization by *H. pylori*, probiotics can prevent *H. pylori* infection in germ-free mice (49). In our study, it seems that due to simultaneous exposure of *H. pylori* and LAB to AGS, competition between them to bind to the host cells may be produced, which is one of the inhibitory mechanisms. 

## Conclusion

Treatment of *H. pylori* using synthetic compounds is associated with several problems such as the high cost of medications, post-treatment bacterial resistance, and adverse side effects. Therefore, exploration of some safer and non-chemical anti-*H. pylori *compounds are becoming important as an alternative therapy against *H. pylori *infections. Probiotic bacteria can inhibit *H. pylori* by immunological and non-immunological mechanisms. Our study reviewed the non-immunological mechanisms that showed the anti*- H. pylori* effect of probiotics bacteria depends on the strain of *H. pylori* and LAB. As lactobacillus can reduce the growth of *H. pylori*, inhibit the activity of its urease, and reduce adhesion *H. pylori* to cell line, they can play an important role in preventing colonization of stomach by *H. pylori*.

## References

[1] Gorbach SL (2000). Probiotics and gastrointestinal health. Am J Gastroenterol.

[2] Islam SU (2016). Clinical uses of probiotics. Medicine (Baltimore).

[3] Diaconu S, Predescu A, Moldoveanu A, Pop CS (2017). Fierbințeanu-Braticevici C Helicobacter pylori infection: old and new. J Med Life.

[4] Figura N, Marano L, Moretti E, Ponzetto A (2016). Helicobacter pylori infection and gastric carcinoma: Not all the strains and patients are alike. World J Gastrointest Oncol.

[5] Backert S, Neddermann M, Maubach G, Naumann M (2016). Pathogenesis of Helicobacter pylori infection. Helicobacter.

[6] De Francesco V, Bellesia A, Ridola L, Manta R, Zullo A (2017). First-line therapies for Helicobacter pylori eradication: a critical reappraisal of updated guidelines. Ann Gastroenterol.

[7] Papastergiou V, Georgopoulos SD, Karatapanis S (2014). Treatment of Helicobacter pylori. World Gastroenterol.

[8] Smith SM, OMorain C, McNamara D (2014). Antimicrobial susceptibility testing for Helicobacter pylori in times of increasing antibiotic resistance. World J Gastroenterol.

[9] Ayala G, Escobedo-Hinojosa WI, de la Cruz-Herrera CF, Romero I (2014). Exploring alternative treatments for Helicobacter pylori infection. World J Gastroenterol.

[10] Kasra -Kermanshahi R, Rezai P (2015). Probiotics and Prebiotics in medicine and dentistry. Iran J Med Microbiol.

[11] McNulty C, Owen R, Tompkins D, Hawtin P, McColl K, Price A (2002). Helicobacter pylori susceptibility testing by disc diffusion. J Antimicrob Chemother.

[12] Falsaﬁ T, Mobasheri F, Nariman F & Najaﬁ M (2004). Susceptibilities to different antibiotics of Helicobacter pylori Strains isolated from Patients at the Pediatric Medical Center of Tehran, Iran. J Clin Microbiol.

[13] Mishra KK, Srivastava S, Grag A, Ayyagari A (2006). Antibiotic Susceptibility of Helicobacter pylori clinical isolates: Comparative evaluation of disk-diffusion and E-test methods. Curr Microbiol.

[14] Wu H, Shi XD, Wang HT, Liu JX (2000). Resistance of Helicobacter pylori to metronidazole, tetracycline and amoxicillin. J Antimicrob Chemother.

[15] Torre J, Camorlinga-Ponce G, Pérez-Pérez A, Madrazo M, Dehesa G, Gonzales V (2001). Increasing multidrug resistance in Helicobacter pylori strains isolated from children and adults in Mexico. J Clin Microbiol.

[16] Sgouras D, Maragkoudakis P, Petraki K, Martinez-Gonzalez B, Eriotou E (2004). In vitro and In vivo Inhibition of Helicobacter pylori by Lactobacillus casei Strain Shirota. Appl Environ Microbiol.

[17] Beessa LJ, Correia DM, Cellini L, Azevedo N, Rocha I (2012). Optimization of culture conditions to improve Helicobacter pylori growth in Ham’s F-12 medium by response surface methodology. Int J Immunopathol Pharmacol.

[18] Mobley HL, Cortesia MJ, Rosenthal L, Jones B (1988). Characterization of urease from Campylobacter pylori. J Clin Microbiol.

[19] Mustafa S, Husaini A, Hipolito N, Hasnain H, Nurshikin S, Hairul AR (2016). Application of response surface methodology for optimizing process parameters in the production of amylase by Aspergillus flavus NSH9 under solid state fermentation. Braz arch boil technol.

[20] Chen X, Liu XM, Tian F, Zhang Q, Zhang HP, Zhang H (2012). Antagonistic activities of lactobacilli against Helicobacter pylori growth and infection in human gastric epithelial cells. JFST.

[21] Ryan KA, Daly P, Li Y, Hooton C, O’Toole PW (2008). Strain-speciﬁc inhibition of Helicobacter pylori by Lactobacillus salivarius and other lactobacilli. J Antimicrob Chemother.

[22] Takeda S, Takeshita M, Matsusaki T, Kikuchi Y, Send-ayush C, Oyunsuren T (2015). In vitro and in vivo anti-Helicobacter pylori activity of probiotics isolated from Mongolian Dairy Products. JFST.

[23] Klerk N, Maudsdotter L, Gebreegziabher H, Saroj S, Eriksson B (2016). Lactobacilli reduce Helicobacter pylori attachment to host gastric epithelial cells by inhibiting adhesion gene expression. Infect Immun.

[24] McNulty CA, Lasseter G, Shaw I, Nichols T, D’Arcy S, Lawson AJ (2012). Is Helicobacter pylori antibiotic resistance surveillance needed and how can it be delivered?. Aliment Pharmacol Ther.

[25] Megraud FH (2004). pylori antibiotic resistance: prevalence, importance, and advances in testing. Gut.

[26] De Francesco V, Giorgio F, Hassan C, Manes G, Vannella L, Panella C (2010). Worldwide H. pylori antibiotic resistance: a systematic review. J Gastrointestin Liver Dis.

[27] Talebi Bezmin Abadi A, Ghasemzadeh A, Taghvaei T, Mobarez AM (2012). Primary resistance of Helicobacter pylori to levofloxacin and moxifloxacine in Iran. Intern Emerg Med.

[28] Khademi F, Poursina F, Hosseini E, Akbari M, Ghasemian H (2015). Helicobacter pylori in Iran: A systematic review on the antibiotic resistance. Iran J Basic Med Sci.

[29] Saniee P, Hosseini F, Kadkhodaei S, Siavoshi F, Khalili-Samani S (2018). Helicobacter pylori multidrug resistance due to misuse of antibiotics in Iran. Arch Iran Med.

[30] Zhang M, Qian W, Qin Y, He I, Zhou Y (2015). Probiotics in Helicobacter pylori eradication therapy: A systematic review and meta-analysis. World J Gastroenterol.

[31] Patel A, Shah N, Prajapati Jb (2014). Clinical application of probiotics in the treatment of Helicobacter pylori infection-A brief review. J Microbiol Immu & infect.

[32] Matjaz H, Rok O (2015). Are probiotics useful in Helicobacter pylori eradication?. World J Gastroenterol.

[33] Losurdo G, Cubisino R, Barone M, Principi M, Leandro G, Ierardi E (2018). Probiotic monotherapy and Helicobacter pylori eradication: A systematic review with pooled-data analysis. World J Gastroenterol.

[34] Francavilla R1, Polimeno L, Demichina A, Maurogiovanni G, Principi B, Scaccianoce G (2014). Lactobacillus reuteri strain combination in Helicobacter pylori infection: a randomized, double-blind, placebo-controlled study. J Clin Gastroenterol.

[35] García A, Navarro K, Sanhueza E, Pineda S, Pastene E, Quezada M (2017). Characterization of Lactobacillus fermentum UCO-979C, a probiotic strain with a potent anti- Helicobacter pylori activity. Electronic J Biotechnology.

[36] Johnson-Henry K, Mitchell D, Avitzur Y, Galindo-Mata E, Jones NL, Sherman PM (2004). Probiotics reduce bacterial colonization and gastric inflammation in H pylori-infected mice. Dig Dis Sci.

[37] Kasra-Kermanshahi R, Mobarak-Qamsari E (2015). Inhibition effect of lactic acid bacteria against food born pathogen, Listeria monocytogenes. Appl Food Biotechnol.

[38] Pacifico L, Osborn J-F, Bonci E, Romaggioli S, Baldini R, Chiesa C (2014). Probiotics for the treatment of Helicobacter pylori infection in childelen. World J Gastroenterol.

[39] Rutherford S, Bassler B (2012). Bacterial quorum sensing: Its role in virulence and possibilities for its control. Cold Spring Harb Perspect Med.

[40] Hathroubi S, Servetas SL, Windham I, Merrell DS, Ottemann KM (2018). Helicobacter pylori biofilm formation and its potential role in pathogenesis. Microbiol Mol Biol Rev.

[41] Aiba Y, Suzuki N, Kabir MA, Takagi A, Koga Y (1998). Lactic acid mediated suppression of Helicobacter pylori by the oral administration of Lactobacillus salivarius as a probiotic in a gnotobiotic murine model. World J Gastroenterol.

[42] Lin W, Wu C, Fang TJ, Guo J, Huang S, Lee M (2011). Anti-Helicobacter pylori activity of fermented milk with lactic acid bacteria. J Sci Food Agric.

[43] Ryan KA, O’Hara A, Van Pijkeren J, Douillard F, O’Toole P (2009). Lactobacillus salivarius modulates cytokine induction and virulence factor gene expression in Helicobacter pylori. J Med Microbiol.

[44] Midolo PD, Lambert JR, Hull R, Luo F, Grayson ML (1995). In vitro inhibition of Helicobacter pylori NCTC11637 by organic acids and lactic acid bacteria. J Appl Bacteriol.

[45] Coconnier MH, Lievin V, Hemery E, Servin AL (1998). Antagonistic activity against Helicobacter pylori infection in vitro and in vivo by human Lactobacillus acidophilus strain LB. Appl Environ Microbiol.

[46] Stingl K, Uhlemann E, Deckers-Hebestreit G, Schmid R, Bakker E, Altendorf K (2001). Prolonged survival and cytoplasmic pH homeostasis of Helicobacter pylori at pH 1. Infect Immun.

[47] Goudarzi L, Kasra Kermanshahi R, Moosavi-Nejad Z, Soltan Dalla M (2017). Evaluation of antimicrobial activity of probiotic Lactobacillus strains against growth and urease activity of Proteus spp. JMB.

[48] Mukai T, Asasaka T, Sato E, Mori K, Matsumoto M, Ohori H (2002). Inhibition of binding of Helicobacter pylori to the glycolipid receptors by probiotic Lactobacillus reuteri. FEMS Immunol Med Microbiol.

[49] Kabir AM, Aiba Y, Takagi A, Kamiya S, Miwa T, Koga Y (1997). Prevention of Helicobacter pylori infection by lactobacilli in a gnotobiotic murine model. Gut.

